# Relapsing Polychondritis Following Alopecia Areata

**DOI:** 10.1155/2010/623158

**Published:** 2010-06-23

**Authors:** John C. Starr, Nidhika Taneja, George W. Brasher

**Affiliations:** ^1^Division of Allergy and Immunology, Department of Internal Medicine, Scott and White Memorial Hospital, Scott, Sherwood, and Brindley Foundation, 2401 South 31st Street, Temple, TX 76508, USA; ^2^Division of Nephrology, Department of Internal Medicine, The Texas A&M Health Sciences Center College of Medicine, Temple, TX, USA

## Abstract

A case of alopecia areata followed by relapsing polychondritis is presented. Similar cases from the literature are reviewed and speculation about the relationship of these diseases is offered. Although the occurrence of these diseases together could be coincidental, an association seems immunologically plausible. Thus, relapsing polychondritis might be an unusual systemic manifestation of alopecia areata.

## 1. Introduction

Alopecia areata affects 1% to 2% of the general population [1]. It has been associated with a number of autoimmune diseases, including thyroid disorders, vitiligo, and possibly atopic dermatitis [2]. Case reports have suggested an association with myasthenia gravis, diabetes mellitus, Addison disease, and even common variable immunodeficiency, amongst others [3, 4]. Twice previously, alopecia areata and relapsing polychondritis have been reported in the same patient [5, 6] We wish to report a third case.

## 2. Case Report

**Figure 1:**
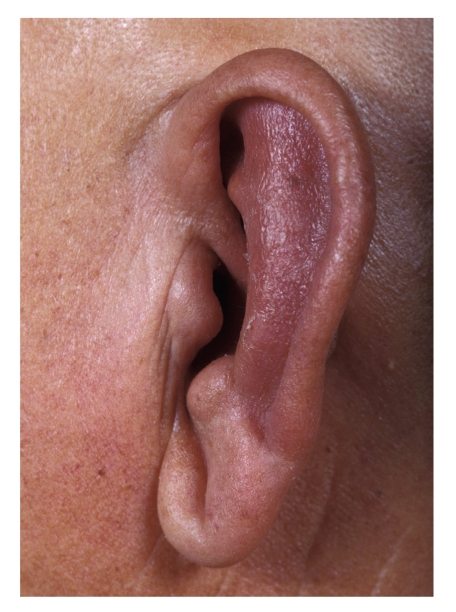
Ear inflammation with sparing of ear lobe.

**Figure 2:**
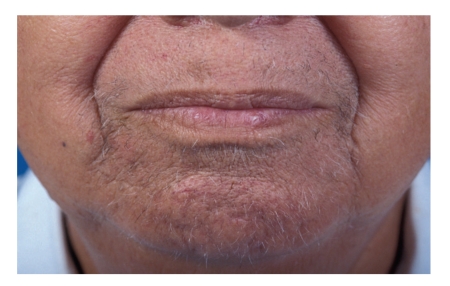
Chin whiskers after prednisone therapy.

A 71-year-old retired welder with a 28-year history of alopecia universalis developed poison ivy on the right forearm. Three weeks later, after complete healing of the poison ivy, the patient noted pain, redness, and tenderness of his right ear. The earlobe was spared. Shortly thereafter, the patient's nose became red and tender, and his left ear developed similar changes (Figure 1). He was treated with antibiotics with no improvement. Because of severe, persistent inflammation of the cartilage of both ears and his nose, a diagnosis of relapsing polychondritis was considered. One week later, prednisone therapy was begun at a dose of 40 mg per day. Prompt resolution of his signs and symptoms occurred. One month later, as prednisone therapy was being tapered, the patient developed hoarseness and a severe sore throat. Rigid telescopy in the ENT clinic found marked swelling of the arytenoids and false vocal cords. Corticosteroids were increased with resolution of his symptoms. Still later, iritis of the left eye occurred. The patient's eye symptoms responded promptly to an increase in corticosteroid therapy. Interestingly, after six weeks of prednisone 30 mg per day, the patient's beard regrew and he shaved for the first time in 28 years! (Figure 2) There was no regrowth of hair on other parts of his body, including the scalp, axillae, eyelashes, or perineal area. Eleven months into the patient's illness, he developed profound sensorineural hearing loss in both ears. An autoimmune basis for his complaints was suspected. There was no history of fever, weight loss, prior similar symptoms, wheezing, or shortness of breath. The physical examination was unremarkable.

Routine laboratory studies that were normal or within the expected range included complete blood count, comprehensive metabolic profile, urinalysis, prostate-specific antigen, thyroid stimulating hormone, c and p antineutrophilic cytoplasmic antibodies, and protein-electrophoresis with immunoglobulins. Sedimentation rate was 43 mm/hr and the antimicrosomal antibody test was positive at 1 : 400 dilution. Pulmonary function tests showed a forced expiratory volume of 78% of predicted and the vital capacity was 71% of predicted, and the carbon monoxide diffusion test was normal. An antibody type II collagen was strongly positive at 65 eu/mL (positive greater than 25 eu/mL). These findings were felt consistent with relapsing polychondritis. A chest roentgenogram was normal.

## 3. Discussion

Alopecia areata is considered a systemic disease, as tissues other than hair follicles are involved [7]. Nail changes include pits, ridges, and reddening of the lunulae. Eye changes have included defects in the retinal pigmentary epithelium [8]. Our patient and the two previously cited cases might suggest that relapsing polychondritis could be yet another systemic manifestation of alopecia areata. In each case, long-term alopecia areata preceded the onset of relapsing polychondritis. Two of the three patients had a particularly severe form of alopecia areata, that is, alopecia universalis. The third patient had recalcitrant alopecia areata (Table 1).

In alopecia areata, the immune response is directed at the hair follicles and helper lymphocytes are thought to play an important role in pathogenesis [3]. Malgouries et al. have surmised that the helper t-cell infiltration of the hair bulb correlates with loss of chondroitin sulfate, an important proteoglycan in both hair follicles and cartilage [9]. In point of fact, an important early finding in relapsing polychondritis is loss of proteoglycans in cartilage [10]. Additionally, a cell-mediated immune response to proteoglycans has been reported in relapsing polychondritis [11]. One could envision a scenario in which lymphocytes, initially active against chondroitin sulfate in the hair follicle, later attack chondroitin sulfate in ear cartilage and perhaps other tissues. It should be noted that chondroitin sulfate helps maintain immune privilege in the hair follicle.

Two weeks prior to the onset of relapsing polychondritis our patient developed poison ivy on his right forearm. The area improved with conservative therapy. As in relapsing polychondritis, both helper and suppressor t-cells are important in the pathogenesis of allergic contact dermatitis [12]. We could not, however, identify literature support for hapten formation between urushiol and proteoglycans. Thus, the relationship between the illness we describe and allergic contact dermatitis remains unknown.

Our patient had a regrowth of beard hair following corticosteroid therapy. The first patient reported with relapsing polychondritis following alopecia areata, a 57-year-old woman, had no regrowth of scalp or body hair following prednisolone therapy. The second previously reported patient (a 13-year-old girl) had conventional alopecia areata, followed by relapsing polychondritis. She had a variable response to prednisone therapy but a more lasting response to a combination of cotrimoxazole treatment and prednisone. Her alopecia areata disappeared with this therapy. Interestingly, in a mouse model, androgens offer resistance to alopecia areata and estrogens promote susceptibility to alopecia areata [13]. Conceivably, our patient's beard regrowth may have been influenced by an androgen milieu in the beard area.

In summary, we present a third case of relapsing polychondritis following long standing alopecia areata. This rare association seems immunologically plausible. As noted by Tobin, both cell-mediated immunity and humoral immunity are likely important in alopecia areata [7]. Likewise, it is possible that there are multiple “targets” of any immune response [7]. As suggested by Tosti et al., damage to retinal pigmentary epithelium might follow an immune attack on hair follicle melanocytes [8]. In a similar vein, relapsing polychondritis could follow a long-term immune assault on a hair follicle proteoglycan, chondroitin sulfate, which is also abundant in ear cartilage and other cartilage-bearing tissues.

## Figures and Tables

**Table 1:** Summary of cases.

Patient	Age	Alopecia universalis	Years of alopecia areata prior to relapsing polychondritis	Hair regrowth with steroids	Onset followed illness	References
1 (female)	56	Yes	14 years	no	no	Kronborg I. J. 1981
2 (female)	13	No	unknown	yes	tonsillitis	Rozin A. P. 2003
3 (male)	71	Yes	28	Beard only	Poison ivy	Starr J. C. 2010
